# Effect of Simulated Toothbrushing on Surface Roughness, Color Stability, and Gloss of Two Single-Shade Composite Resins

**DOI:** 10.3390/ma19081523

**Published:** 2026-04-10

**Authors:** Zeynep Hale Keles, Vasfiye Isik, Soner Sismanoglu

**Affiliations:** 1 Department of Restorative Dentistry, Faculty of Dentistry, Istanbul Atlas University, Atlas Vadi Kampüsü, Anadolu Cd. No 40 Kağıthane, 34408 Istanbul, Türkiye; 2 Department of Endodontics, Faculty of Dentistry, Istanbul University-Cerrahpaşa, 34098 Istanbul, Türkiye; vasfiye.isik@iuc.edu.tr; 3 Department of Restorative Dentistry, Faculty of Dentistry, Istanbul University-Cerrahpaşa, 34098 Istanbul, Türkiye

**Keywords:** single-shade composite resin, simulated toothbrushing, surface roughness, color stability, gloss

## Abstract

This in vitro study evaluated and compared the surface roughness, color stability, and gloss of two single-shade composite resins after simulated toothbrushing, and investigated the correlations among these parameters. Twenty disk-shaped specimens (*n* = 10 per group) were prepared from two single-shade composite resins (Material A and Group B) and subjected to simulated toothbrushing up to 15,000 cycles. Surface roughness (Ra) was measured at baseline and after 5000, 10,000, and 15,000 cycles. Color parameters (CIE Lab*) and gloss (60°) were measured at baseline and after 15,000 cycles. Color change was calculated using the CIEDE2000 formula (ΔE_00_). Data were analyzed using two-way mixed ANOVA, *t*-tests, and Pearson correlation analysis (α = 0.05). Both materials showed progressive increases in surface roughness. Material B exhibited significantly higher Ra values than Material A from 10,000 cycles onward (*p* < 0.01). After 15,000 cycles, Material B demonstrated significantly greater color change (ΔE_00_: 2.21 ± 0.18 vs. 1.48 ± 0.13; *p* < 0.001), exceeding the acceptability threshold (ΔE_00_ = 1.8), while Material A remained clinically acceptable. Material B also showed greater gloss reduction (60% vs. 35%; *p* < 0.001). Strong correlations were found between surface roughness and both gloss change (r = −0.919) and color change (r = 0.826). Material A demonstrated greater resistance to surface degradation and better preservation of optical properties compared to Material B. Surface roughness was identified as the common underlying factor driving both color instability and gloss reduction in single-shade composites. Clinical Significance: Not all single-shade composites perform equally under mechanical aging. Clinicians should consider the filler technology and long-term surface stability when selecting single-shade composite resins for clinical use.

## 1. Introduction

Resin-based composites are firmly established as the most commonly used direct restorative materials in contemporary dentistry, largely due to their esthetic versatility, conservative preparation design, and broad clinical applicability [1]. Yet, one of the persistent challenges in restorative practice is achieving a satisfactory color match between the composite and the adjacent tooth structure [2]. With conventional multi-shade systems, clinicians must choose from an extensive shade range, a process that is subjective by nature and prone to error, particularly under inconsistent lighting conditions or when operator experience varies [3]. Managing a wide inventory of shade-specific composites can also increase material costs, and less frequently used shades may expire before they are fully utilized [4]. Single-shade universal composite resins were developed to address these issues. These materials are designed to match all 16 VITA Classical shades with a single composite, eliminating the shade selection step altogether and streamlining the restorative workflow [5]. Unlike conventional composites that rely on pigments and dyes for color reproduction, single-shade systems utilize optical phenomena, including structural color generation, light scattering, and blending effects, to adapt chromatically to the surrounding tooth [3,6].

Omnichroma (Material A; Tokuyama Dental, Tokyo, Japan) was the first commercially available single-shade composite to use what the manufacturer terms “Smart Chromatic Technology.” The system is based on uniformly sized, spherical SiO_2_-ZrO_2_ supra-nano fillers of 260 nm diameter. When ambient light enters the composite, these fillers selectively amplify red-to-yellow wavelengths, the spectral components common to all natural tooth shades, via diffraction and interference [5]. The resulting structural color blends with the reflected color of the surrounding teeth through additive color mixing, enabling shade adaptation without the use of pigments [6,7]. Since its introduction, Material A has been the subject of several investigations evaluating its shade-matching ability, color stability, and surface properties, with generally favorable outcomes reported across a range of clinical and laboratory conditions [8]. Zenchroma (Material B; President Dental, Germany) takes a different approach. It is a light-curing microhybrid composite with ultrafine radiopaque filler particles ranging from 0.005 to 3.0 µm (75 wt%/53 vol%), consisting of glass powder and silicon dioxide in a Bis-GMA/UDMA/TEGDMA matrix. Although Material B also claims to replicate all 16 VITA Classical shades, its underlying shade-matching mechanism has received comparatively less scientific characterization than that of Material A [4]. Its filler system is also notably different, relying on a broad particle size distribution rather than monodisperse spherical fillers, which may have implications for both wear behavior and the preservation of optical properties under functional conditions [9,10].

The surface characteristics of a composite restoration have a direct impact on its clinical performance and longevity. Surface roughness, in particular, influences plaque accumulation, susceptibility to staining, and even patient comfort, with a clinically relevant Ra threshold of 0.2 µm proposed as the level below which bacterial adhesion no longer decreases meaningfully [11]. For single-shade composites, however, surface integrity carries an additional dimension of clinical importance. Because these materials rely on precisely controlled light–filler interactions to generate structural color, any disruption of the surface layer has the potential to compromise not only roughness and gloss but also the shade-matching mechanism itself [5]. Color stability is another key concern, as discoloration has been identified as one of the common reasons for replacing anterior composite restorations [12]. The CIEDE2000 color difference formula is now widely accepted for quantifying color changes in dental research, with perceptibility (PT_00_ = 0.8) and acceptability (AT_00_ = 1.8) thresholds established through a multicenter visual assessment study [13]. Gloss contributes significantly to the perceived esthetics of a restoration and is tightly linked to surface topography; as a surface becomes rougher, incoming light is scattered diffusely rather than reflected specularly, resulting in a dull appearance [10]. The interplay among these three parameters, roughness, color, and gloss, is therefore particularly relevant for single-shade composites, where surface degradation may simultaneously affect mechanical, optical, and chromatic performance [5,14].

Toothbrushing, while essential for oral hygiene, subjects the restoration surface to repeated mechanical wear. Simulated brushing studies have consistently demonstrated that abrasion progressively increases surface roughness and reduces gloss, though the extent of these changes varies considerably between materials and is governed primarily by filler architecture and matrix composition [10]. For conventional composites, the clinical consequences of surface degradation are largely limited to increased plaque retention and reduced esthetics. For single-shade composites, however, the stakes may be higher. Because these materials depend on controlled light interactions with their filler particles to produce structural color, brushing-induced surface disruption has the potential to alter the spectral behavior of the filler system, compromising not only roughness and gloss but also the chromatic adaptation that defines their clinical utility [5]. Material A’s monodisperse 260 nm supra-nano spherical fillers and Material B’s broadly distributed microhybrid filler system represent fundamentally different approaches to achieving single-shade performance, and it is reasonable to expect that their responses to mechanical aging will differ accordingly [4,6]. Yet, direct comparisons between these two materials under standardized brushing conditions have not been reported.

Despite growing interest in single-shade composites, relatively few studies have examined how different formulations respond to mechanical aging in terms of surface and optical properties. Several investigations have addressed color matching, polishability, and color stability following staining or thermocycling protocols [15,16,17], but the specific effects of simulated toothbrushing on the relationship between roughness, color, and gloss in these materials remain underexplored. Direct comparisons between Material A and Material B, two composites built on the same clinical premise but with substantially different filler technologies, are particularly scarce.

The aim of this study was therefore to compare the surface roughness, color stability, and gloss of Material A and Material B after simulated toothbrushing. Roughness was measured at baseline and after 5000, 10,000, and 15,000 brushing cycles; color and gloss were assessed at baseline and following 15,000 cycles. Correlations among these three parameters were also evaluated. The null hypothesis was that there would be no significant difference between the two materials in any of the tested parameters.

## 2. Materials and Methods

### 2.1. Specimen Preparation

This in vitro study evaluated two single-shade resin composites: Omnichroma (Material A) and Zenchroma (Material B). Detailed composition, filler content, and manufacturer information for each material are given in Table 1.

Sample size was determined using G*Power software (version 3.1.9.7, Heinrich Heine University Düsseldorf, Germany). Surface roughness (Ra) served as the primary outcome variable. Based on pilot data and the observed variance in Ra values, a large effect size (f = 0.60) was estimated for the material × brushing cycle interaction in a repeated-measures design. With α = 0.05, power (1 − β) = 0.80, two groups, and four measurement time points, the analysis indicated a minimum of 8 specimens per group. To account for possible specimen loss and improve reliability, 10 specimens per group were prepared, yielding a total of 20.

Disk-shaped specimens (10 mm diameter × 2 mm thickness) were fabricated by placing each composite into a cylindrical stainless-steel mold. A polyethylene terephthalate (PET) strip and glass slide were pressed over the surface to ensure flatness and standardization. Polymerization was carried out for 20 s per surface using a light-emitting diode (LED) curing unit (Valo Grand, Ultradent, South Jordan, UT, USA) through the PET strip and glass slide in direct contact with the specimen surface. The irradiance output was verified prior to each curing session using a dental radiometer (Coxo, Foshan, China) to ensure a minimum of 1000 mW/cm^2^. Both the top and bottom surfaces were light-cured to ensure thorough conversion. Following polymerization, specimens were removed from the molds and sequentially polished using the complete four-step Sof-Lex disc system (coarse, medium, fine, and superfine; 3M ESPE, St. Paul, MN, USA) under light intermittent pressure with continuous water irrigation. A fresh set of discs was used for each specimen. Each disc was applied using a slow-speed handpiece at approximately 10,000 rpm for 20 s per specimen surface to achieve a uniform surface finish. They were then cleaned in an ultrasonic bath containing distilled water for 5 min and stored in distilled water at 37 °C for 24 h before baseline measurements were taken. The same specimens were used for all roughness, color, and gloss assessments throughout the experiment.

### 2.2. Simulated Toothbrushing Procedure

Specimens were brushed using a programmable toothbrushing simulation apparatus (MF-100, Mod Dental, Esetron Smart Robotechnologies, Ankara, Turkey) under standardized conditions. The device operated with a circular motion pattern (15 mm diameter) at a speed of 40 mm/s. Soft polyamide-bristle toothbrush heads were mounted and applied with a constant vertical load of 200 g. A new toothbrush head was used for each specimen. A brushing slurry was prepared by mixing a fluoride toothpaste (Colgate Total, Colgate-Palmolive Co., Guanzhou, China; RDA ≈ 70) and distilled water at a 1:2 ratio by weight; the slurry was freshly prepared for each specimen. The 15,000-cycle protocol was selected to simulate approximately one to one and a half years of clinical toothbrushing, based on published estimates of daily brushing stroke frequency [18,19]. Specimens were removed from the apparatus at each measurement interval (5000, 10,000, and 15,000 cycles), cleaned in an ultrasonic bath, and repositioned randomly for the subsequent brushing phase.

Surface roughness was recorded at four time points: baseline, 5000, 10,000, and 15,000 cycles. Color and gloss were measured at baseline and after the full 15,000-cycle protocol. Between each measurement interval, specimens were rinsed and returned to distilled water storage at 37 °C.

### 2.3. Surface Roughness Measurements

Surface roughness (Ra, µm) was assessed with a contact profilometer (Surtronic S128, Taylor Hobson, Leicester, UK) using a cut-off length of 0.8 mm and an evaluation length of 4 mm. For each specimen, three readings were taken at random orientations across different surface locations and averaged. Measurements were repeated at baseline and after 5000, 10,000, and 15,000 brushing cycles.

### 2.4. Color and Gloss Measurements

Color was measured with a portable reflectance spectrophotometer (Vita Easyshade V, Vita Zahnfabrik, Bad Säckingen, Germany) using the CIE Lab* system. Specimens were placed on a white background under standardized lighting, and three readings were taken from the center of each specimen and averaged. Color differences between baseline and post-brushing (15,000 cycles) were quantified using the CIEDE2000 formula (ΔE_00_).

Gloss was recorded with a glossmeter (GM-26, Murakami Color Research Laboratory, Tokyo, Japan) at a 60° incidence angle. Three readings per specimen were averaged at each time point. Measurements were taken at baseline and after 15,000 cycles, and the change (ΔGloss) was calculated as the difference between the two.

### 2.5. Statistical Analysis

All analyses were conducted in SPSS (Version 31, IBM Corp., Armonk, NY, USA). Normality was checked with the Shapiro–Wilk test. Surface roughness data were evaluated using two-way mixed ANOVA (material × time), with Bonferroni-corrected pairwise comparisons for post hoc testing. Color change and gloss data were compared using paired *t*-tests (within-group) and independent samples *t*-tests (between-group). Pearson correlation coefficients were calculated to explore associations among Ra, ΔE_00_, and ΔGloss. Significance was set at *p* < 0.05.

## 3. Results

### 3.1. Surface Roughness

**Figure 1:**
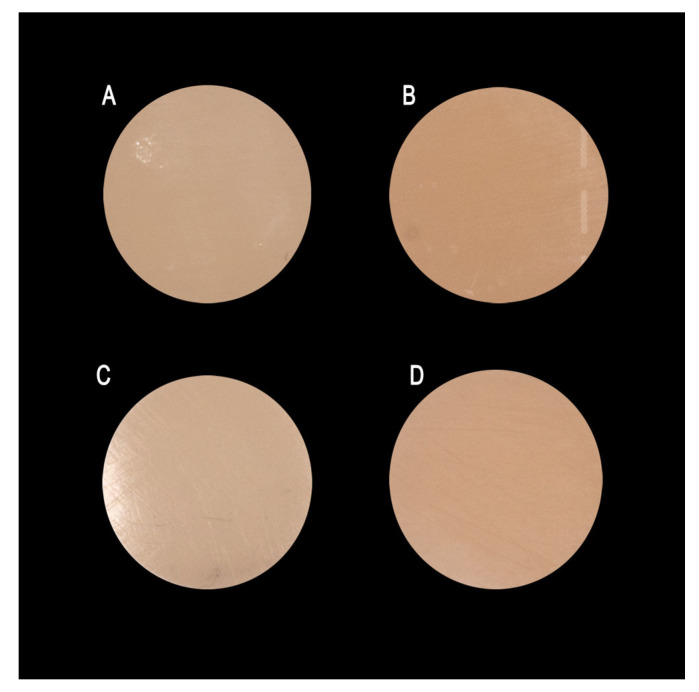
Representative images of Material A (**A**,**C**) and Material B (**B**,**D**) specimens at baseline (**A**,**B**) and after 15,000 simulated toothbrushing cycles (**C**,**D**). Note the visible surface alterations and loss of gloss in both materials following brushing simulation, with more pronounced changes observed in Material B specimens. Specimen dimensions: 10 mm diameter × 2 mm thickness.

Ra data were normally distributed across all groups (Shapiro–Wilk, *p* > 0.05). Two-way mixed ANOVA showed significant main effects for both material (F(1,18) = 9.917, *p* = 0.006, η^2^p = 0.355) and time (F(3,54) = 419.113, *p* < 0.001, η^2^p = 0.959), as well as a significant material × time interaction (F(3,54) = 14.992, *p* < 0.001, η^2^p = 0.454). Greenhouse–Geisser correction was applied because sphericity was violated (ε = 0.602). Baseline and post-brushing specimen images are shown in Figure 1.

**Figure 2:**
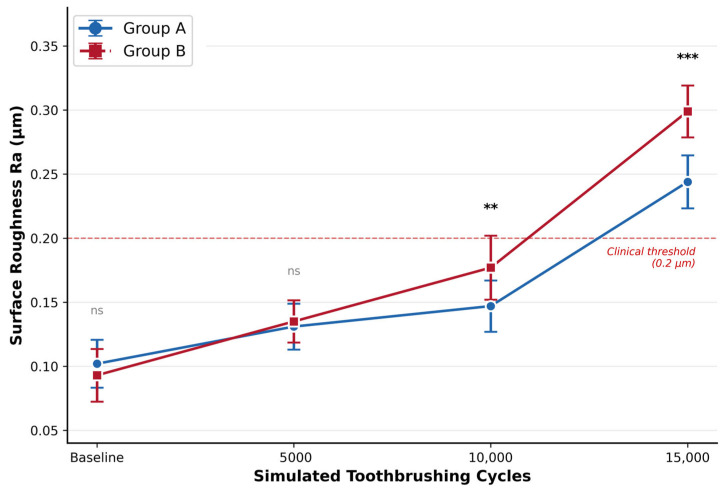
Mean surface roughness (Ra, µm) of Material A and Material B composite resins at baseline and after 5000, 10,000, and 15,000 simulated toothbrushing cycles. Error bars represent standard deviation (*n* = 10). The red dashed line indicates the clinically relevant threshold for bacterial adhesion (Ra = 0.2 µm). Statistical significance between materials at each time point was determined by independent samples *t*-test (ns: not significant; ** *p* < 0.01; *** *p* < 0.001). Two-way mixed ANOVA revealed significant effects of material (*p* = 0.006), time (*p* < 0.001), and material × time interaction (*p* < 0.001).

The two materials started with comparable roughness at baseline (Material A: 0.102 ± 0.019 µm; Material B: 0.093 ± 0.021 µm; *p* = 0.320), and no significant difference was present at 5000 cycles either (*p* = 0.610). The divergence began at 10,000 cycles, where Material B showed significantly higher Ra than Material A (*p* = 0.008), and this gap widened further at 15,000 cycles (*p* < 0.001) (Table 2, Figure 2). Bonferroni-corrected comparisons confirmed that both materials roughened progressively over time. However, the pattern differed: Material A showed no significant change between 5000 and 10,000 cycles (*p* = 0.845), suggesting a temporary plateau, while Material B increased significantly at every consecutive interval (*p* < 0.001).

By 15,000 cycles, both composites had exceeded the 0.2 µm clinical threshold for bacterial adhesion, with Material B reaching 0.299 ± 0.020 µm and Material A 0.244 ± 0.021 µm. The significant interaction effect confirms that the two materials degraded at different rates, with Material B following a steeper and more continuous roughening trajectory.

### 3.2. Color Change and Gloss

**Figure 3:**
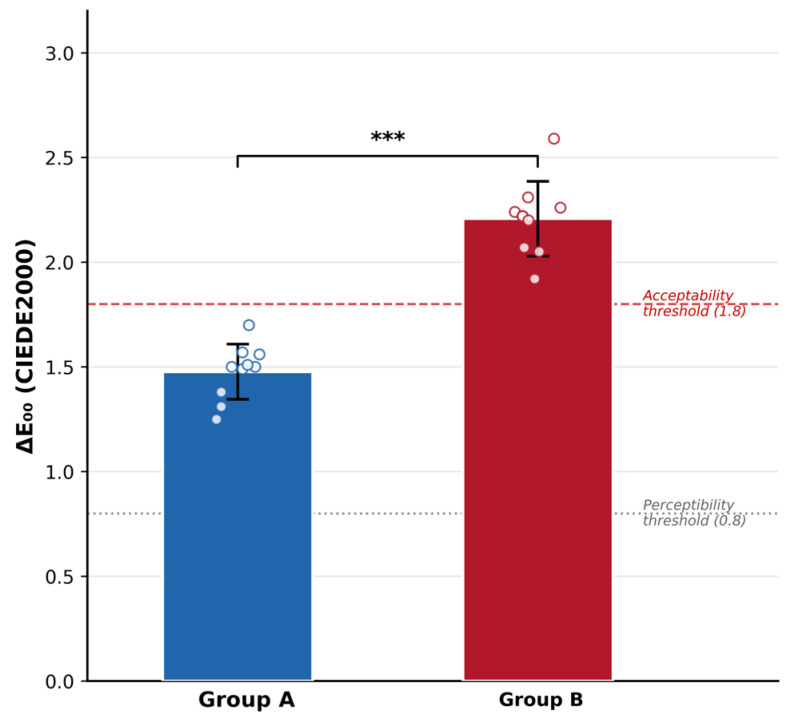
Color change (ΔE_00_) of Material A and Material B composite resins after 15,000 simulated toothbrushing cycles. Bars represent mean values with error bars indicating standard deviation. Individual specimen data points are displayed as circles (*n* = 10). The gray dotted line indicates the perceptibility threshold (ΔE_00_ = 0.8) and the red dashed line indicates the acceptability threshold (ΔE00 = 1.8). *** *p* < 0.001 (independent samples *t*-test).

All color data passed normality testing (Shapiro–Wilk, *p* > 0.05). After 15,000 brushing cycles, Material B showed significantly greater overall color change than Material A (ΔE_00_: 2.21 ± 0.18 vs. 1.48 ± 0.13; *p* < 0.001, Cohen’s d = 4.66) (Table 3, Figure 3). Clinically, this distinction is meaningful. Material A’s mean ΔE_00_ stayed below the acceptability threshold of 1.8, while Material B’s exceeded it, suggesting that the color shift in Material B restorations would likely be noticeable and potentially unacceptable to patients. The mean between-group differences at 15,000 cycles were as follows: Ra, 0.055 µm (95% CI: 0.036 to 0.074); ΔE_00_, 0.73 (95% CI: 0.58 to 0.88); ΔGloss, −23.33 GU (95% CI: −31.99 to −14.67). All confidence intervals excluded zero, confirming the statistical significance of the observed differences.

Looking at individual coordinates, both materials became lighter after brushing, as reflected by positive ΔL* values (Material A: 2.35 ± 0.21; Material B: 2.83 ± 0.30; *p* < 0.001). The more notable differences emerged in the chromatic axes. Material B shifted substantially toward green (Δa*: −1.09 ± 0.12 vs. −0.09 ± 0.10; *p* < 0.001, Cohen’s d = 9.09) and blue (Δb*: −0.94 ± 0.11 vs. −0.51 ± 0.14; *p* < 0.001, Cohen’s d = 3.49). The very large effect sizes, particularly for Δa*, highlight a pronounced chromatic alteration in Material B that was largely absent in Material A.

Baseline gloss values were similar between groups (Material A: 85.75 ± 8.66 GU; Material B: 89.20 ± 5.69 GU; *p* = 0.306), indicating that initial polishing conditions were comparable. Both materials lost gloss significantly after 15,000 cycles (paired *t*-test, *p* < 0.001 for both), but the extent of loss was markedly different. Material A retained a post-brushing gloss of 55.28 ± 3.65 GU, losing roughly 35% of its initial value (ΔGloss: −30.47 ± 9.03 GU). Material B dropped to 35.40 ± 8.46 GU, a reduction of approximately 60% (ΔGloss: −53.80 ± 9.41 GU; *p* < 0.001, Cohen’s d = 2.53). In practical terms, Material A preserved nearly two-thirds of its original gloss, while Material B retained less than half.

### 3.3. Correlations Between Surface Roughness and Optical Properties

**Figure 4:**
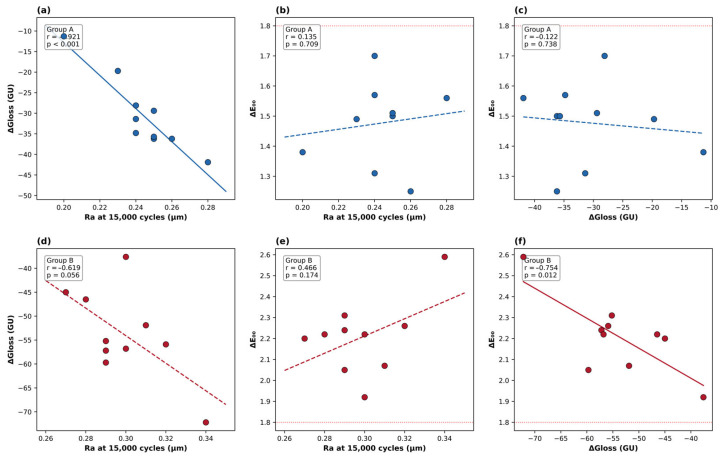
Within-group Pearson correlation analyses for Material A (**a**–**c**) and Group B (**d**–**f**). (**a**,**d**) Ra at 15,000 cycles versus gloss change; (**b**,**e**) Ra at 15,000 cycles versus color change (ΔE_00_); (**c**,**f**) gloss change versus color change. Solid regression lines indicate statistically significant correlations (*p* < 0.05); dashed lines indicate non-significant correlations. The red dotted line in panels (**b**,**c**,**e**,**f**) marks the acceptability threshold (AT, ΔE_00_ = 1.8). *n* = 10 per group.

Pearson correlation analyses were performed within each material group separately (*n* = 10) to avoid potential inflation of correlation coefficients from between-group differences (Figure 4). Within Material A, a strong negative correlation was observed between Ra at 15,000 cycles and gloss change (r = −0.921, *p* < 0.001), indicating that rougher specimens lost more gloss. Within Material B, this relationship was moderate and borderline significant (r = −0.619, *p* = 0.056). In contrast, the correlation between Ra and ΔE_00_ was weak and non-significant within both groups (Material A: r = 0.135, *p* = 0.709; Material B: r = 0.466, *p* = 0.175). The correlation between gloss change and color change was non-significant in Material A (r = −0.122, *p* = 0.738) but strong and significant in Material B (r = −0.754, *p* = 0.012).

## 4. Discussion

This study set out to compare how two single-shade composites, Material A and Material B, respond to simulated toothbrushing in terms of surface roughness, color stability, and gloss, and to explore the relationships among these parameters. Across all three outcomes, Material B performed significantly worse than Material A. The null hypothesis was therefore rejected.

Surface roughness is among the most clinically important properties of a composite restoration. The widely cited Ra threshold of 0.2 µm [11] marks the point below which further smoothing does not meaningfully reduce bacterial adhesion. In the present study, both materials crossed this threshold by 15,000 cycles, but Material B (0.299 ± 0.020 µm) did so to a considerably greater degree than Material A (0.244 ± 0.021 µm). These values are broadly consistent with the roughness ranges reported for nanohybrid composites after extended brushing simulation [15,20,21], though direct comparisons with other single-shade systems remain scarce [8,22]. What was perhaps more informative than the final values was the degradation trajectory. The significant material × time interaction (*p* < 0.001, η^2^p = 0.454) showed that the two composites roughened in distinctly different ways. Material A appeared to reach a plateau between 5000 and 10,000 cycles, suggesting a phase of relative stability, before roughening again. Material B, on the other hand, showed continuous, uninterrupted roughening at every interval. From a clinical standpoint, this suggests that Material B surfaces may deteriorate more rapidly during the early stages of service, a concern that is particularly relevant given that single-shade composites are frequently selected for anterior restorations where surface quality directly affects esthetic outcomes [5,23].

These differences likely reflect the underlying filler architecture of each material. Material A’s uniform, spherical SiO_2_-ZrO_2_ supra-nano fillers (260 nm) are reported to create a homogeneous distribution within the resin matrix [6], which is expected to produce more even wear. Under such conditions, filler and matrix may erode at comparable rates, resulting in a relatively smooth surface. The uniformity and monodisperse nature of these fillers, as characterized in optical and color-matching studies [6], is analogous to the filler architecture of nanofilled composites containing nanoclusters, where fine, well-distributed particles have been shown to minimize differential wear and maintain surface integrity after abrasion [10]. SEM-based investigations have confirmed that composites with spherical, uniformly distributed fillers maintain smoother surfaces after brushing, whereas those with irregularly shaped particles of varying sizes exhibit filler plucking and greater surface disruption [24]. Similarly, experimental studies using uniform spherical fillers have demonstrated a direct correlation between filler particle size and post-brushing roughness and gloss retention, with finer fillers consistently outperforming larger ones [25]. This is consistent with a recent investigation showing that Material A exhibited the lowest roughness among several composite types, a finding attributed to its uniform filler dispersion [22].

Material B, while also marketed as a single-shade system, has a considerably broader filler size range (0.005–3.0 µm) and different particle morphology. This heterogeneity may cause uneven wear during abrasion: softer matrix regions are removed more readily, while larger or harder filler particles resist or are plucked out, both scenarios producing a rougher topography [10,21]. The role of filler size distribution in determining abrasion behavior has been well documented; composites with larger or irregularly shaped filler particles tend to exhibit higher post-polishing roughness compared to those with finer, more uniformly distributed fillers [10,26]. Similar material-dependent roughening patterns have been observed in brushing studies on other composites [19], and Ra values in the range of 0.14–0.49 µm after 20,000 brushing cycles have been reported for nanohybrid systems [20], consistent with our findings. Taken together, the evidence suggests that the monodisperse supra-nano filler system of Material A confers a greater in abrasion resistance compared to the heterogeneous microhybrid filler architecture of Material B, and that this difference becomes increasingly apparent with prolonged mechanical challenge [10,21].

Both materials showed color changes above the perceptibility threshold (ΔE_00_ = 0.8), meaning the shifts would be clinically detectable. The critical difference, though, is where each material falls relative to the acceptability threshold (ΔE_00_ = 1.8), a benchmark derived from a multicenter study with 175 observers [13]. Material A’s mean ΔE_00_ (1.48 ± 0.13) remained below this cutoff, suggesting that while the color change is perceivable, it would likely be tolerated clinically. Material B (2.21 ± 0.18) exceeded it, raising concerns about esthetic acceptability over time. It is worth noting that color stability in single-shade composites has broader clinical implications than in conventional systems, as any chromatic shift may raise concerns about the long-term intrinsic color stability of these materials [5,16]. These findings align with earlier work showing Material A to have greater color stability compared to various composite formulations after staining challenges [8,27]. A study that tested five single-shade composites, including both Material A and Material B, under combined staining, brushing, and thermal cycling also reported material composition as a key determinant of color change [16]. Moreover, recent investigations have confirmed that the color stability of single-shade composites is not uniform across commercial products, with considerable differences observed depending on filler type, resin matrix chemistry, and the specific shade-matching technology employed [5,7,16].

The individual color coordinates offer some additional insight. Both materials became lighter after brushing (positive ΔL*), a phenomenon that has been frequently reported in studies evaluating color changes in abraded or aged composite surfaces [21,27]. As the resin matrix wears away, inorganic filler particles become more exposed at the surface, increasing light scattering and producing a brighter appearance. An association between surface roughness and lightness changes has been noted in studies on resin composites, where rougher surfaces tend to scatter more light and appear brighter [20]. Where the two materials diverged more strikingly was in the chromatic coordinates. Material B exhibited a pronounced green shift (Δa*; Cohen’s d = 9.09) and a blue shift (Δb*; Cohen’s d = 3.49), effect sizes that are exceptionally large. The near-absence of chromatic shift in Material A (Δa: −0.09) compared to the substantial green shift in Material B (Δa: −1.09) is particularly noteworthy, as it suggests that Material A’s monodisperse supra-nano filler system may better preserve its spectral behavior after surface wear, whereas Material B ‘s heterogeneous filler architecture may be more susceptible to optical disruption [5,23]. We speculate that these shifts may relate to surface-level disruption of the filler–light interactions that characterize single-shade composites. Since these materials depend on precisely controlled optical phenomena to generate structural color [5], surface degradation could alter spectral behavior in ways that go beyond what conventional pigment-based composites would exhibit [5,16].

Gloss followed a similar pattern to roughness and color. Starting from equivalent baselines (*p* = 0.306), the two materials diverged substantially after brushing. Material A retained about 65% of its initial gloss, while Material B held onto only about 40%. For single-shade composites intended primarily for esthetically demanding anterior restorations, this magnitude of gloss loss is clinically concerning, as gloss is one of the first properties perceived by patients and clinicians when evaluating restoration quality [5,23]. A comprehensive brushing simulation study showed that surface gloss was in fact the parameter that best discriminated between dental materials after brushing, with a strong roughness–gloss correlation [18]. A recent study evaluating surface properties after 80,000 brushing strokes similarly confirmed that filler characteristics are the primary determinant of gloss retention, with materials containing smaller filler particles exhibiting less gloss reduction over time [28]. Post-brushing gloss values as low as 10.6 GU have been reported for some composites [29], illustrating how severe the loss can be depending on the material. Recent work on single-shade restorative composites has similarly confirmed that gloss reduction after polishing is material-dependent and closely tied to filler characteristics [22]. The greater gloss reduction in Material B is consistent with its rougher post-brushing surface, as increased surface irregularities promote diffuse scattering at the expense of specular reflection. More surface irregularities mean more diffuse scattering and less specular reflection. The fact that Material A maintained nearly two-thirds of its original gloss while Material B retained less than half further supports the role of a uniform, monodisperse filler system in preserving optical surface quality under mechanical aging [5,10].

The within-group correlation analyses revealed a more nuanced picture than a pooled approach would suggest. The relationship between surface roughness and gloss loss was strong and significant within Material A (r = −0.921, *p* < 0.001) and moderate within Material B (r = −0.619, *p* = 0.056). This is consistent with the well-established physical mechanism linking surface irregularities to diffuse light scattering and reduced specular reflection [10,18], and aligns with roughness–gloss associations in the range of r = 0.7–0.9 reported across various composite formulations [10,21]. Similar interrelationships have been reported in brushing simulation studies on various dental materials [18], and more recently in work on single-shade restorative composites [22]. In contrast, the correlation between surface roughness and color change was weak and non-significant within both material groups (Material A: r = 0.135; Material B: r = 0.466), indicating that color change was not directly driven by surface roughness within a given material. This finding suggests that the color instability observed in Material B is related to material-specific factors, likely its heterogeneous filler architecture and the resulting differences in optical behavior, rather than being a direct consequence of surface roughening. For single-shade composites, this distinction is particularly relevant because these materials generate color at or near the surface through filler–light interactions [5]. The fact that Material B exhibited both greater roughening and greater color change does not necessarily imply a causal link between the two; rather, both outcomes may stem from the same underlying material characteristic, namely a broadly distributed filler system that is simultaneously more vulnerable to mechanical disruption and less capable of preserving its optical function under wear [5,16]. These findings reinforce the importance of filler architecture as the central determinant of both mechanical and optical durability in single-shade composites [22].

It should also be considered that the oral environment introduces additional factors that may modulate the surface and optical changes observed under purely mechanical brushing conditions. In vivo, dental composite surfaces are rapidly covered by an acquired salivary pellicle, which forms a protective protein layer that may attenuate brushing-induced wear and slow surface roughening [30]. Conversely, pH cycling and exposure to acidic dietary components can accelerate hydrolytic degradation of dimethacrylate-based resin matrices through acid-catalyzed ester bond cleavage, potentially exacerbating surface deterioration and amplifying the performance differences observed between materials with distinct matrix compositions [31]. Similarly, the moderate abrasivity of the toothpaste used in this study (RDA ≈ 70) was selected to standardize the brushing challenge and ensure comparability with the existing literature. Toothpastes with higher abrasivity, such as whitening formulations, could accelerate surface degradation and potentially amplify the performance differences between materials, whereas lower-abrasivity formulations may attenuate them. The influence of toothpaste abrasivity on the relative behavior of single-shade composites warrants further investigation. Dietary chromogens may introduce additional color changes through extrinsic staining, compounding the intrinsic chromatic shifts reported in this study. Thermocycling-induced thermal stresses could promote filler–matrix debonding, as repeated temperature fluctuations have been shown to degrade the silane coupling interface and cause filler particle plucking from the resin matrix [32,33]. This mechanism may be particularly relevant for composites with heterogeneous filler systems, where differential thermal expansion between particles of varying size could generate interfacial stress concentrations. The combined effect of these factors in vivo may therefore either mitigate or intensify the differences reported here, and the results of this study should be interpreted with these considerations in mind.

From a clinical perspective, the results carry a clear message. Single-shade composites have gained traction because they simplify shade selection and reduce chair time [3], but our data show that not all of these materials age in the same way. Material A’s supra-nano spherical filler system appears to be associated with greater resistance in resisting surface degradation and maintaining optical integrity over time. The performance gap between the two materials became statistically significant at the 10,000-cycle mark and continued to widen, which, if extrapolated to clinical use, could translate into meaningful esthetic differences within the first few years of service.

Several limitations should be noted. This was an in vitro study, and the oral environment involves additional factors such as salivary pellicle, pH cycling, dietary staining, and temperature fluctuations that were not simulated here. Color and gloss were measured only at baseline and after 15,000 cycles; intermediate assessments would have provided a more detailed picture of how optical properties evolve over time, though our approach is consistent with most published brushing-simulation protocols. We compared only two materials without including a conventional multi-shade composite as a control, which limits the broader interpretability of the results. Surface roughness was assessed using two-dimensional contact profilometry (Ra), and three-dimensional areal parameters such as Sa were not evaluated. While 3D non-contact profilometry would provide a more comprehensive surface characterization, the Ra parameter was selected to allow direct comparison with the clinically established 0.2 µm threshold and the existing literature. Finally, the absence of SEM analysis means that our discussion of degradation mechanisms remains partly speculative. Additionally, the compositional data used in this study were based on manufacturer-provided specifications, and independent material characterization (e.g., EDS, XRD, or FTIR) was not performed. Future studies should consider combining brushing with thermocycling and staining protocols, testing a wider range of single-shade composites, and incorporating microscopic surface analysis to better characterize the degradation process.

## 5. Conclusions

Within the limitations of this in vitro study, the following conclusions were drawn:

1. Both single-shade composite resins exhibited a progressive increase in surface roughness with increasing brushing cycles; however, Material B demonstrated significantly greater roughening than Material A from 10,000 cycles onward.
2. Material B showed significantly greater color change (ΔE_00_) than Material A, exceeding the clinical acceptability threshold, whereas Material A remained within the clinically acceptable range.
3. Both materials exhibited significant gloss loss after simulated toothbrushing; however, Material B showed approximately twice the gloss reduction compared to Material A.
4. Strong correlations were observed between surface roughness and optical property changes, indicating that surface degradation is the common underlying factor driving both color instability and gloss reduction.
5. Material A demonstrated greater overall resistance to surface degradation and more effective preservation of optical properties compared to Material B under simulated toothbrushing conditions.

## Figures and Tables

**Table 1:** Batch number, Composition, filler ratio, and manufacturer details of the resin composites tested.

Resin Composites	Compositions	Filler Ratio	Batch Number
Zenchroma; President, Allershausen, Germany	Glass powder, di-UDMA, silicon dioxide, Bis-GMA, tetrameth ylene dimethacrylate. Microhybrid and ultrafine fillers	75% by weight 53% by volume	2025005643
Omnichroma; Tokuyama, Tokyo, Japan	UDMA, TEGDMA, Uniform sized supra-nano spherical filler (260 nm SiO_2_-ZrO_2_)	79% by weight 68% by volume	204M35

Bis-GMA; bisphenol A-glycidyl methacrylate, TEGDMA; triethylene glycol dimethacrylate, UDMA; urethane dimethacrylate.

**Table 2:** Surface roughness (Ra, µm) of composite resins at different simulated toothbrushing cycles (mean ± SD).

Material	Baseline	5000 Cycles	10,000 Cycles	15,000 Cycles	*p* (Time) *
Material A	0.102 ± 0.019 ^d^	0.131 ± 0.018 ^c^	0.147 ± 0.020 ^bc^	0.244 ± 0.021 ^a^	<0.001
Material B	0.093 ± 0.021 ^d^	0.135 ± 0.016 ^c^	0.177 ± 0.025 ^b^	0.299 ± 0.020 ^a^	<0.001
*p* (material) **	0.320	0.610	0.008	<0.001	

Two-way mixed ANOVA: Material effect F(1,18) = 9.917, *p* = 0.006, η^2^p = 0.355; Time effect F(3,54) = 419.113, *p* < 0.001, η^2^p = 0.959; Material × Time interaction F(3,54) = 14.992, *p* < 0.001, η^2^p = 0.454. Greenhouse–Geisser correction was applied (ε = 0.602). Different lowercase superscript letters (a, b, c, d) within the same row indicate statistically significant differences between time points (Bonferroni-corrected pairwise comparisons, *p* < 0.05). * Repeated-measures ANOVA within each material group. ** Independent samples *t*-test between materials at each time point.

**Table 3:** Color change parameters (ΔL, Δa, Δb, and ΔE_00_) and gloss values (GU) after 15,000 simulated toothbrushing cycles (mean ± SD).

Parameter	Material A	Material B	*p*-Value	Cohen’s d	Significance
**Color Change**					
ΔL	2.35 ± 0.21	2.83 ± 0.30	<0.001	1.87	***
Δa	−0.09 ± 0.10	−1.09 ± 0.12	<0.001	9.09	***
Δb	−0.51 ± 0.14	−0.94 ± 0.11	<0.001	3.49	***
ΔE_00_	1.48 ± 0.13	2.21 ± 0.18	<0.001	4.66	***
**Gloss (GU)**					
Baseline	85.75 ± 8.66	89.20 ± 5.69	0.306	0.47	ns
After 15,000 cycles	55.28 ± 3.65	35.40 ± 8.46	<0.001	3.05	***
ΔGloss	−30.47 ± 9.03	−53.80 ± 9.41	<0.001	2.53	***

Color change values represent the difference between baseline and 15,000 brushing cycles. ΔE_00_ was calculated using the CIEDE2000 formula. Perceptibility threshold: ΔE_00_ = 0.8; Acceptability threshold: ΔE_00_ = 1.8. Paired *t*-test (Baseline vs 15,000 cycles): Material A *p* < 0.001; Material B *p* < 0.001. Between-group comparisons were performed using independent samples *t*-test. Effect size reported as Cohen’s d. *** *p* < 0.001; ns: not significant.

## Data Availability

The data presented in this study are available on request from the corresponding author due to their use in ongoing research and potential future publications.
